# Cross‐country variance in facial emotion recognition in presymptomatic and symptomatic behavioral variant frontotemporal dementia: Insights from the GENFI and ReDLat consortia

**DOI:** 10.1002/alz.70741

**Published:** 2025-10-14

**Authors:** Liset de Boer, Lize C. Jiskoot, Harro Seelaar, John C. van Swieten, Agustin Ibanez, Marcelo Maito, Sol Fittipaldi, Julie F. H. De Houwer, Tine Swartenbroekx, Pam A. Boesjes, Rhian S. Convery, Eve Ferry‐Bolder, Phoebe Foster, Arabella Bouzigues, Lucy Chisman‐Russell, Esther van den Berg, Janne Papma, Sanne Franzen, Renelle Bourdage, James B. Rowe, Barbara Borroni, Daniela Galimberti, Pietro Tiraboschi, Mario Masellis, Elizabeth Finger, Robert Laforce, Caroline Graff, Alexander Gerhard, Raquel Sanchez‐Valle, Alexandre Mendonça, Fermin Moreno, Matthis Synofzik, Rik Vandenberghe, Simon Ducharme, Isabelle Le Ber, Johannes Levin, Thibaud Lebouvier, Benedetta Nacmias, Markus Otto, Christopher R. Butler, Isabel Santana, Maxime Bertoux, M. Carmela Tartaglia, Jonathan D. Rohrer, Jackie M. Poos, the GENFI Consortium

**Affiliations:** ^1^ Alzheimer Center Department of Neurology Erasmus University Medical Center Rotterdam the Netherlands; ^2^ Latin American Brain Health Institute (BrainLat) Universidad Adolfo Ibañez, Peñalolén Santiago de Chile Chile; ^3^ Global Brain Health Institute (GBHI) Trinity College Dublin Dublin Ireland; ^4^ Dementia Research Centre Department of Neurodegenerative Disease UCL Queen Square Institute of Neurology London UK; ^5^ Department of Internal Medicine Erasmus University Medical Center Rotterdam the Netherlands; ^6^ Université Paris Cité Laboratoire Mémoire Cerveauet Cognition (UR7536) Institut de Psychologie Boulogne‐Billancourt France; ^7^ Department of Clinical Neurosciences and Cambridge University Hospitals NHS Trust University of Cambridge Cambridge UK; ^8^ Department of Clinical and Experimental Sciences University of Brescia Viale Europa Brescia Italy; ^9^ Molecular Markers Laboratory IRCCS Istituto Centro San Giovanni di Dio Fatebenefratelli Brescia Italy; ^10^ Fondazione Ca’ Granda IRCCS Ospedale Policlinico Milan Italy; ^11^ Dept. of Biomedical, Surgical and Dental Sciences University of Milan Milan Italy; ^12^ Fondazione IRCCS Institute Neurologico Carlo Besta Milan Italy; ^13^ Sunnybrook Health Sciences Centre Sunnybrook Research Institute University of Toronto Toronto Canada; ^14^ Department of Clinical Neurological Sciences University of Western Ontario London ON Canada; ^15^ Clinique Interdisciplinaire de Mémoire Département des Sciences Neurologiques, and Faculté de Médecine Université Laval Rue Québec (Qc) QC Canada; ^16^ Department of Neurobiology Care Sciences and Society Center for Alzheimer Research Division of Neurogeriatrics, Bioclinicum Karolinska Institutet Solna Sweden; ^17^ Division of Neuroscience and Experimental Psychology Wolfson Molecular Imaging Centre University of Manchester Withington Manchester UK; ^18^ Alzheimer's disease and Other Cognitive Disorders Unit, Neurology Service, Hospital Clínic, Institut d'Investigacións Biomèdiques August Pi I Sunyer University of Barcelona Barcelona Spain; ^19^ Faculty of Medicine University of Lisbon Lisbon Portugal; ^20^ Cognitive Disorders Unit Department of Neurology Donostia Universitary Hospital, Donostia San Sebastian Spain; ^21^ Department of Neurodegenerative Diseases Hertie‐Institute for Clinical Brain Research and Center of Neurology University of Tübingen Tübingen Germany; ^22^ Laboratory for Cognitive Neurology Department of Neurosciences KU Leuven Leuven Belgium; ^23^ Department of Psychiatry Douglas Mental Health University Institute and Montreal Neurological Institute McGill University Montréal Québec Canada; ^24^ Sorbonne Université Paris Brain Institute – Institut du Cerveau – ICM Inserm U1127, CNRS UMR 7225, AP‐HP ‐ Hôpital Pitié Paris France; ^25^ Department of Neurology LMU University Hospital, LMU Munich Germany; ^26^ German Center for Neurodegenerative Diseases site Munich Munich Germany; ^27^ Munich Cluster for Systems Neurology (SyNergy) Munich Germany; ^28^ Univ. Lille, Inserm, CHU Lille, Lille Neuroscience & Cognition Inserm, Rue Michel Polonowski Lille France; ^29^ Department of Neurofarba University of Florence Firenze Italy; ^30^ Department of Neurology University of Ulm Ulm Germany; ^31^ Nuffield Department of Clinical Neurosciences Medical Sciences Division University of Oxford Oxford UK; ^32^ The George Institute for Global Health School of Public Health Imperial College London London UK; ^33^ University Hospital of Coimbra (HUC) Neurology Service Faculty of Medicine University of Coimbra Coimbra Portugal; ^34^ Tanz Centre for Research in Neurodegenerative Diseases University of Toronto Toronto ON Canada

**Keywords:** cultural diversity, facial emotion recognition, frontotemporal dementia, presymptomatic, social cognition

## Abstract

**INTRODUCTION:**

We investigated international differences in facial emotion recognition (FER) across stages of frontotemporal dementia (FTD). Previous studies may have missed early decline by combining data and masking variations in FER across countries.

**METHODS:**

An FER test was administered to 159 individuals with behavioral variant FTD, 521 presymptomatic pathogenic variant carriers, and 583 controls from 16 countries of residence. Linear mixed models assessed age, sex, education, and country effects on FER. Voxel‐based morphometry examined neural correlates across countries.

**REULTS:**

Country accounted for 18%–18.3% of FER variance in presymptomatic carriers and controls and 9.9% in individuals with behavioral variant of FTD (bvFTD). Cross‐country differences interacted with the effects of sex, age, and education. Neural correlates involving the frontal lobe and basal ganglia were identified in individuals with bvFTD, but no cross‐country differences were found.

**DISCUSSION:**

These results underscore the need for culturally sensitive FER tools in research and clinical practice, especially as global multinational clinical trials emerge.

**Highlights:**

- Performance on a test for facial emotion recognition (FER) varies between countries.
- The percentage of variance is lower in the behavioral variant of frontotemporal dementia (bvFTD) compared to presymptomatic pathogenic variant carriers and healthy controls.
- Cross‐country differences interacted with the effects of sex, age, and education.
- There were no differences in brain correlates of FER across countries.

## 1 INTRODUCTION

Frontotemporal dementia (FTD) is a neurodegenerative disorder affecting primarily the frontal and temporal brain lobes.^1^ Individuals with FTD often experience impairments of social skills, which can have devastating effects on their interpersonal relationships and functioning in daily life.^2^, ^3^, ^4^, ^5^ Family members frequently report loss of social and personal conduct, personal warmth, empathy and sympathy, and interest in daily‐life activities, causing a higher caregiver burden than in other types of dementia.^6^ The cognitive processes that underlie such skills are generally grouped together within the term ‘social cognition’ and include several abilities, including recognition of others’ emotions, “theory of mind” (ToM, the ability to infer and understand others’ thoughts and beliefs), understanding of social norms, and moral reasoning.^3^, ^4^, ^7^ Given that significant behavioral and emotional changes are often among the earliest symptoms reported by caregivers—sometimes even years prior to diagnosis^8^—socio‐cognitive assessment has the potential to enhance early diagnosis and facilitate timely intervention.

FTD is inherited in an autosomal dominant manner in up to ∼30% of cases.^9^ Studying genetic forms of FTD provides the opportunity to identify clinical changes before symptom onset.^10^ Large prospective cohort studies have been set up across the world to investigate the preclinical stage of FTD and have demonstrated gene‐specific cognitive changes in attention, executive function, language, and memory; however, findings are less consistent for social cognition.^11^, ^12^, ^13^, ^14^ This is a striking discrepancy considering that a deficit in social cognition is a core criterion for FTD diagnosis.^15^ Multiple clinical trials testing disease‐modifying treatments for presymptomatic FTD pathogenic variant carriers have started. However, a major challenge is the lack of sensitive tools for social cognition to identify the optimal time window for treatment initiation and to track disease progression in the earliest stages of the disease.

A confounding factor in defining social cognition in presymptomatic FTD and/or in detecting decline over time could be insufficient cross‐cultural validity of traditional measures for social cognition that are used in global cohort studies (e.g., the *Genetic Frontotemporal Initiative* (GENFI)^10^ and the *Multi‐Partner Consortium to Expand Dementia Research in Latin America* (ReDLat).^16^ Bourdage et al.^17^ highlighted limitations in the global suitability of existing assessments, noting that many tools fail to capture social cognition across diverse cultural and linguistic contexts due to a lack of adequate adaptation or translation. For example, the Ekman 60 Faces Test^18^ assesses solely facial emotion recognition (FER) of white individuals, and previous studies frequently lacked detailed information about diagnostic accuracy, reliability, and validity in different cultures. Furthermore, linguistic research has demonstrated that emotional conceptual understanding varies across different languages, which is likely to influence FER.^19^, ^20^ Taken together, these problems raise the risk of bias, diagnostic error, and insensitivity to neural correlates of abnormal social recognition.

Variance in social cognition across cultures has already been shown in other studies.^21^, ^22^, ^23^ In addition, a recent study from Quesque et al.^24^ examined the influence of country on FER and ToM in a cognitively healthy population across 12 countries. They found that after controlling for age, education, and sex, cross‐country differences accounted for more than 20% of the variance in FER. Moreover, it is conceivable that country interacts with the effects of sex, age, and education, as observed in a study where culture influenced the relationship between age and working memory performance.^25^ These findings have significant consequences for international collaborations interested in clinical group contrasts, as Quesque and colleagues conclude that, if a test is validated in one country, it cannot automatically be used across the world for assessment purposes.^24^

With the emergence of international multicenter cohort studies on FTD aiming to harmonize research across these individual cohorts, it is crucial to examine culture‐dependent factors influencing the available clinical data. This study examined the variation in FER performance across 16 countries in Europe and Latin America within the GENFI and ReDLat consortia and explored which underlying factors (e.g., age, sex, education, gray matter [GM] volume, and GM) are associated with cross‐country differences.

**RESEARCH IN CONTEXT**

1. **Systematic review**: The authors reviewed the literature using public sources (e.g., PubMed). Facial emotion recognition (FER) has been used worldwide in clinical settings as a measure of social cognition and has been studied systematically in familial frontotemporal dementia (FTD). However, FER in familial FTD has yet to be examined in a cross‐cultural context.
2. **Interpretation**: This study emphasizes the need to account for cultural differences in social cognition. We highlight the complex interplay of country of residence on effects of age, sex, and education on FER performance, suggesting these factors cannot be assumed to impact participants across all countries in the same way.
3. **Future directions**: Results from this study provide new insights and guidance for future research, such as increasing the inclusion of more data across different countries, as well as including additional aspects of culture, such as social determinants of educational differences and socioeconomic status.

## 2 METHODS

### 2.1 Participants

We included a total of 1163 participants from 16 different countries (Table 1) from the GENFI cohort^10^ and the ReDLat cohort^16^ (individuals with the behavioral variant of FTD [bvFTD]), *n* = 159; presymptomatic pathogenic variant carriers, *n* = 421; controls, *n* = 583). We excluded other FTD phenotypes, as impairments in social cognition are a defining feature of bvFTD, and sample sizes in other phenotypes were small.

**TABLE 1:** Demographic data and mean FER performance per country and clinical group.

	Controls (*n* = 583)					Presymptomatic (*n* = 421)					bvFTD (*n* = 159)					
Country	*n*	Age (years)	Education (years)	Sex ratio (F%)	FER	*n*	Age (years)	Education (years)	Sex ratio (F%)	FER	*n*	Age (years)	Education (years)	Sex ratio (F%)	FER	Statistical differences
TOTAL	583	51.42 (14.64)	14.35 (4.25)	63.12%	27.77 (4.03)	421	44.30 (11.66)	14.72 (3.37)	58.91%	28.75 (3.21)	159	63.79 (8.82)	12.77 (4.03)	40.48%	19.81 (6.43)	Age: bvFTD > con > pre Education: bvFTD < pre = con Sex: significant FER: bvFTD < pre = con
Argentina	28	45.68 (11.85)	16.11 (3.64)	71.43%	29.18 (3.80)											NA
Belgium	15	41.81 (8.32)	16.67 (2.38)	53.33%	29.87 (2.13)	15	50.43 (11.80)	14.73 (2.34)	66.67%	29.20 (2.27)	7	66.84 (4.21)	14.86 (2.04)	42.86%	18.57 (6.48)	Age: bvFTD > pre = con Education: n.s. Sex: n.s. FER: bvFTD < pre = con
Brazil	41	57.49 (13.93)	15.27 (4.04)	70.73%	27.56 (3.44)											NA
Canada	69	42.80 (13.27)	15.03 (2.46)	60.87%	28.46 (2.68)	55	41.44 (12.42)	15.71 (2.57)	56.36%	27.96 (3.31)	15	63.94 (7.69)	14.53 (3.18)	40.00%	20.80 (5.80)	Age: bvFTD > pre = con Education: n.s. Sex: n.s. FER: bvFTD < pre = con
Chile	30	64.33 (10.22)	15.73 (3.07)	80.00%	27.87 (3.62)						8	65.75 (7.69)	15.12 (2.75)	50.00%	22.88 (5.57)	Age: n.s. Education: n.s. Sex: n.s. FER: bvFTD < con
Colombia	22	62.73 (8.41)	15.14 (5.05)	63.64%	26.50 (4.16)						22	67.32 (8.51)	11.68 (5.86)	45.45%	23.45 (5.70)	Age: n.s. Education: bvFTD < con Sex: n.s. FER: bvFTD < con
France	13	41.92 (10.80)	15.77 (2.42)	69.23%	30.08 (4.07)	17	42.31 (8.43)	15.24 (3.13)	58.82%	30.41 (2.60)						All n.s.
Germany	16	40.87 (12.71)	15.25 (2.44)	62.50%	25.75 (2.57)	18	36.94 (12.05)	15.67 (2.28)	55.56%	26.06 (2.62)	10	57.31 (11.96)	12.60 (2.41)	60.00%	16.60 (5.27)	Age: bvFTD > pre = con Education: bvFTD < pre Sex: n.s. FER: bvFTD < pre = con
Italy	42	44.66 (10.49)	12.57 (3.70)	47.62%	28.57 (3.16)	53	45.11 (12.01)	13.49 (3.86)	52.83%	28.58 (3.39)	26	62.07 (11.26)	9.96 (4.13)	30.77%	19.12 (5.77)	Age: bvFTD > pre = con Education: bvFTD < pre Sex: n.s. FER: bvFTD < pre = con
Mexico	21	60.33 (12.87)	16.33 (4.90)	52.38%	24.76 (3.74)											NA
Netherlands	55	50.62 (13.03)	14.11 (3.04)	56.36%	29.24 (3.57)	67	48.63 (12.05)	13.85 (3.20)	67.16%	30.18 (2.69)	10	57.68 (6.10)	14.90 (2.33)	40.00%	22.60 (6.08)	Age: n.s. Education: n.s. Sex: n.s. FER: bvFTD < pre = con
Peru	85	66.00 (7.66)	10.40 (5.70)	76.47%	24.18 (5.13)						22	66.95 (8.02)	13.68 (3.68)	54.55%	19.45 (6.95)	Age: n.s. Education: bvFTD > con Sex: n.s. FER: bvFTD < con
Portugal	17	41.88 (13.24)	13.88 (3.28)	64.71%	27.82 (2.67)	27	44.04 (8.52)	13.70 (3.07)	70.37%	27.19 (3.27)						All n.s.
Spain	44	47.49 (11.69)	16.27 (4.41)	52.27%	30.27 (2.67)	60	45.07 (11.58)	16.12 (4.37)	60.00%	29.32 (3.63)	9	65.29 (7.12)	10.33 (3.16)	33.33%	15.00 (6.02)	Age: bvFTD > pre = con Education: bvFTD < pre = con Sex: n.s. FER: bvFTD < pre = con
Sweden	23	45.20 (12.45)	13.91 (2.35)	56.52%	30.13 (2.46)	22	49.51 (9.81)	14.45 (3.33)	50.00%	29.05 (2.98)						All n.s.
UK	62	46.34 (13.87)	15.27 (2.99)	61.29%	27.85 (3.33)	87	41.36 (10.58)	14.67 (2.80)	55.17%	28.40 (2.76)	30	62.83 (6.86)	13.40 (2.97)	30.00%	18.57 (6.82)	Age: bvFTD > pre = con Education: n.s. Sex: significant FER: bvFTD < pre = con

*Note*: Data are presented as mean (standard deviation).

Abbreviations: bvFTD, behavioral variant of frontotemporal dementia; con, controls; FER, facial emotion recognition; NA, not applicable; n.s., not significant; pre, presymptomatic.

#### 2.1.1 GENFI cohort

Baseline data were included from the seventh GENFI data freeze in which participants from genetic FTD families were recruited between January 2012 and June 2022 in 25 study centers. In this study, we recruited 884 participants from Belgium (*n* = 37), Canada (*n* = 139), France (*n* = 30), Germany (*n* = 40), Italy (*n* = 121), the Netherlands (*n* = 132), Portugal (*n* = 44), Spain (*n* = 113), Sweden (*n* = 45), and the United Kingdom (*n* = 179).

The FER component of the Mini‐Social Cognition and Emotional Assessment (Mini‐SEA)^26^ was administered in a total of 528 FTD pathogenic variant carriers (presymptomatic *n* = 421, symptomatic *n* = 107) and 356 pathogenic‐negative controls. Symptomatic pathogenic variant carriers were diagnosed with bvFTD (62 *C9orf72*, 20 *GRN*, 24 *MAPT*, 1 *TARDBP*) for whom a clinical diagnosis was obtained in a multidisciplinary consensus involving experienced neurologists, neuropsychologists, radiologists, and geriatricians, and had a Clinical Dementia Rating plus National Alzheimer's Coordinating Center Behavior and Language Domains (CDR plus NACC FTLD)^27^ score of ≥1. Diagnosis was based on the current consensus criteria for bvFTD.^15^ Presymptomatic pathogenic variant carriers (182 *C9orf72*, 166 *GRN*, 66 *MAPT*, 1 *TARDBP*, 5 *TBK1*, and 1 *VCP*) did not fulfill the diagnostic FTD criteria and received a CDR plus NACC FTLD score of ≤0.5. 323 controls, 385 presymptomatic pathogenic variant carriers, and 87 individuals with bvFTD had a FER test at baseline and a structural (T1‐weighted) magnetic resonance imaging (MRI) brain scan.

#### 2.1.2 ReDLat cohort

Baseline data were included from the ReDLat study in which individuals with bvFTD and healthy controls were recruited between April 2021 and September 2023. A total of 279 participants were recruited from Argentina (*n* = 28), Brazil (*n* = 41), Chile (*n* = 38), Colombia (*n* = 44), Mexico (*n* = 21), and Peru (*n* = 107). The FER component of the Mini‐SEA was administered to a total of 52 individuals with bvFTD and 227 healthy controls. Diagnosis was based on the current consensus criteria for bvFTD.^15^ Individuals with bvFTD did not undergo genetic testing. 225 controls and 52 individuals with bvFTD had an FER test at baseline and a structural (T1‐weighted) magnetic resonance imaging (MRI) brain scan.

### 2.2 Standard protocol approvals, registrations, and patient consents

This observational study was conducted in accordance with the Strengthening the Reporting of Observational Studies in Epidemiology (STROBE) reporting guidelines. All GENFI and ReDLat sites had local ethical approval for the study, and all participants provided written informed consent. The study was in accordance with the Declaration of Helsinki.

### 2.3 Procedure

All GENFI and ReDLat participants underwent a standardized clinical interview (including the CDR plus NACC FTLD^27^) and comprehensive neurological and neuropsychological assessment covering the major cognitive domains. Most participants underwent laboratory testing (lumbar puncture and/or blood sampling) and structural MRI. The FER test^19^ was included in the standard neuropsychological assessment.

### 2.4 FER test

We used the FER test of the Mini‐SEA, which originally consists of both a test of FER and a test of ToM.^28^ The test of ToM, the faux pas test, was not included in the current study because it was not part of the standard GENFI protocol. The FER test is a 35‐item version of the original Ekman 60 Faces Test,^18^ and contains black and white photos of non‐Latino white faces of 16 men and 19 women. All faces were presented in a fixed random order. For each face, participants were instructed to indicate whether the faces are showing one of the six ‘universal’ emotions (i.e., happiness, anger, sadness, fear, surprise, and disgust) or a neutral expression. The total score (/35) was used in this study, as well as the separate scores per emotion (/5). In the GENFI study, the FER test has been translated into multiple languages (English, Dutch, Italian, French, Spanish, Portuguese, German, and Swedish) through independent translation and back‐translation and has been used in numerous studies to assess social cognition in FTD.^28^, ^29^, ^30^, ^31^ The translations are available on request.

### 2.5 Structural brain imaging and voxel‐based morphometry

GENFI participants underwent volumetric T1‐weighted MRI according to the GENFI imaging protocol on six types of 3T scanners from different vendors: GE Signa, Siemens Magnetom Vida, Siemens Trio, Siemens Skyra, Siemens Prisma, and Philips Achieva. ReDLat participants underwent volumetric T1‐weighted MRI according to the ReDLat imaging protocol on eight different 3T scanners: Siemens Biograph, Siemens Magnetom Lumina, Siemens Magnetom Spectra, Siemens Skyra, Siemens Skyla, GE Signa, Philips Achieva, and Philips Ingenia Elition.

All scans underwent extensive visual quality checks, and images with significant artifacts or incidental brain abnormalities unrelated to bvFTD were excluded from further analysis (*n* = 17). In addition, seven scans of individuals with bvFTD were excluded from further analysis because the sample size was too small (Argentina *n* = 1, Brazil *n* = 2, France *n* = 1, Mexico *n* = 1, Sweden *n* = 2). In total, volumetric T1 scans of 548 controls, 382 presymptomatic pathogenic variant carriers, and 138 individuals with bvFTD were included in the VBM analysis. Images were preprocessed using the VBM Toolbox in Statistical Parametric Mapping 12 (SPM12; www.fil.ion.ucl.ac.uk/spm), running in MATLAB R2021b (Mathworks), and segmented to obtain GM, white matter (WM), and cerebrospinal fluid (CSF) volumes. GM segmentations were modulated, smoothed, and transformed into the Montreal Neurological Institute (MNI) space. Total intracranial volume (TIV, i.e., GM+WM+CSF) was calculated in SPM12 ((www.fil.ion.ucl.ac.uk/spm), running in MATLAB R2021b (Mathworks)).

### 2.6 Statistical analysis

Statistical analyses were performed using R (v4.3.1, R Foundation for Statistical Computing, Vienna, Austria) with the following packages: *lmerTest, ggplot2, dplyr*, and *car*. The significance level was set at *p* < 0.05 (2‐tailed) across all comparisons, and we implemented Bonferroni corrections for multiple testing. Normality was assessed using Q–Q plots. We compared continuous demographic data between groups with *t*‐tests or two‐way analyses of variance and post‐hoc tests based on estimated marginal means. We analyzed sex distribution using chi‐squared tests.

To analyze cross‐country differences per clinical group, we included country as random effect (FER ∼ age + sex + education + (1|country)). To assess differences in FER performance across different countries, we contrasted the AIC values of the linear mixed model described above with a model comprising solely age, sex, and education. In addition, we examined how country interacts with age, sex, and education by including interaction terms in our analysis. To visualize these effects, we generated figures displaying the fixed effects and predicted FER scores for each country, along with 95% confidence intervals (CIs). In the presymptomatic group, we explored whether including genetic subgroup as covariate significantly improved the model. To analyze the potential (confounding) effect of language,^24^ we added language as fixed effect in the model. However, we tested whether language and country were strongly correlated to account for collinearity concerns (based on the Variance Inflation Factor in the *car* package). As the results indicated multicollinearity (i.e., language of administration and country were the same in almost all participants), we continued our analyses with only country as random effect and did not control for language in further analyses. We explored the variance between languages in an additional model where we added language as random effect (FER ∼ age + sex + education + (1|language)).

We then performed an analysis for the variance partitioning coefficients (VPC) to quantify the proportion of variation in the FER performance (total scores and emotion subscores) that is attributable to various levels of the model (different countries). A VPC close to 100% suggests that a significant portion of the total variability is attributed to differences at the specified level for one of the predictors.^24^ We also computed the VPC for each emotion separately to examine any differences within emotions on the variance of the random effects. We analyzed Hedges’ *g* values between every pair of countries for cross‐country comparisons.

The relationship between the FER total score and GM volume, while controlling for the effect of country, was analyzed using multiple regression models. Country is included as covariate in the model by means of dummy variables. Age, sex, years of education, TIV, and scanner type (as some countries had various types of scanners) were also included as covariates. In addition, an interaction between country and FER performance was added to the model to explore potential moderating effects. All comparisons were corrected for a family‐wise error (FWE) rate of 0.05.

## 3 RESULTS

### 3.1 Demographics

Demographic and clinical data are presented in Table 1. In the total cohort, individuals with bvFTD were significantly older than presymptomatic pathogenic variant carriers (β = 19.49) and controls (β = 12.38) [F(2,1160) = 133.8, *p* < 0.001]. Additionally, controls were significantly older than presymptomatic pathogenic variant carriers (β = 7.12). Individuals with bvFTD had fewer years of education compared to both presymptomatic pathogenic variant carriers (β = −1.96) and controls (β = −1.58) [F(2,1160) = 14.65, *p* < 0.001]. Sex distribution differed across groups [χ^2^(2) = 25.50, *p* < 0.001], with a higher percentage of females in presymptomatic pathogenic variant carriers and controls compared to individuals with bvFTD. Across the full sample, FER total scores were lower in individuals with bvFTD compared to presymptomatic pathogenic variant carriers (β = −7.36) and controls (β = −6.78) [F(5,1157) = 169.8, *p* < 0.001].

Demographic data and FER total mean scores per country of the healthy control group, the presymptomatic pathogenic variant carriers, and the individuals with bvFTD are presented in Table 1. In all countries, individuals with bvFTD were significantly older than presymptomatic pathogenic variant carriers and/or controls (all *p* < 0.05), except for Chile, Colombia, France, the Netherlands, Peru, Portugal, and Sweden. Level of education did not significantly differ in most countries, but individuals with bvFTD had a lower level of education in Colombia, Germany, Italy, and Spain compared to presymptomatic pathogenic variant carriers and controls (*p* < 0.05). In addition, individuals with bvFTD from Peru had a higher level of education compared to their healthy controls (*p* < 0.05). Except for the United Kingdom, there were no significant differences in country‐level sex distributions. FER performance was significantly lower in individuals with bvFTD compared to presymptomatic pathogenic variant carriers and/or controls in all countries (all *p* < 0.05).

Significant differences were found between the GENFI and ReDLat cohorts in age [t(109.87) = 3.31, *p* = 0.001], where individuals with bvFTD from the ReDLat cohort were older (mean difference 4.65, 95% CI 1.79–7.51). No significant differences were found between education and sex distribution. In healthy controls, ReDLat participants were older (mean difference 15.52, 95% CI 13.43–17.61) [t(494.58) = 14.70, *p* <#x000A0;0.001) and had fewer years of education (mean difference 1.08, 95% CI 0.37–1.78) [t(334.34) = −2.71, *p* = 0.007) compared to the GENFI cohort (Table S1).

### 3.2 Group‐specific analyses

#### 3.2.1 Healthy controls

**FIGURE 1:**
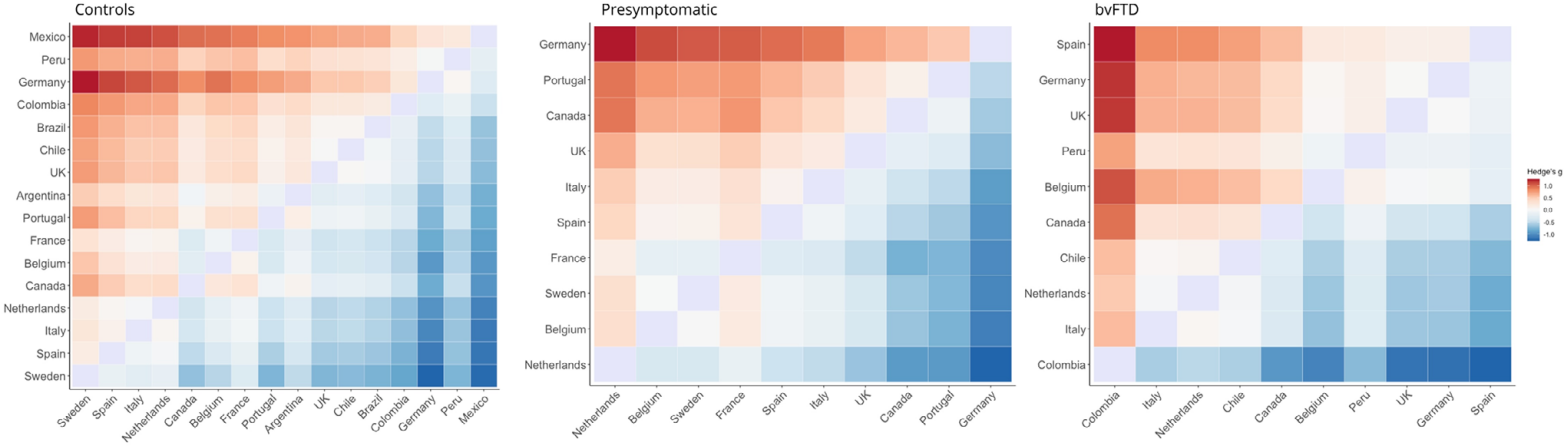
Heatmap of the difference between every pair of countries measured with Hedge's g. Positive values correspond to a better performance of column countries compared to row countries.

The likelihood ratio test statistic between the model with country as random effect and the model comprising solely age, sex, and education was significant [X^2^(1) = 53.81, *p* < 0.001], indicating superior prediction of scores when incorporating the random country effect into the model. The VPC indicated that 18.3% of the variance in FER performance can be attributed to differences among countries after controlling for age, sex, and education. The Hedges’ *g* ranged from *g* = 0.01 (Chile vs. United Kingdom) to *g* = 1.23 (Sweden vs. Germany) (Figure 1). Variance across countries for each emotion (mean and 95% CI) is illustrated in Figure S1. The emotion with the largest effect of country after controlling for age, sex, and education was disgust, VPC = 21.93%, followed by fear, VPC = 7.73%, anger, VPC = 4.85%, sadness, VPC = 4.67%, surprise, VPC = 4.66%, and happiness, VPC = 1.03%. When including language as a random effect in the model instead of country, we observe a lower VPC of 13.5%.

**FIGURE 2:**
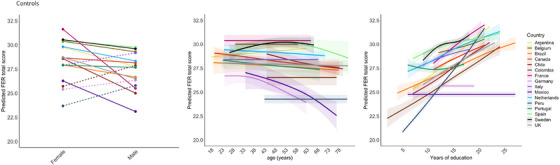
Effects of sex, age, and education per country in healthy controls. Note: Dotted lines indicate a higher predicted FER total score in males compared to females. The effects of age and education on the predicted FER total score are visualized with 95% confidence intervals. FER, facial emotion recognition.

Controlling for the effect of country, sex had an effect on the FER total score, with women having higher scores than men [β = −0.87, *p* = 0.002] (Figure 2). In addition, we found a positive effect of education on the FER total score [β = 0.38, *p* < 0.001]. Age and language did not have a significant effect on FER total scores. There was a significant interaction between country and sex, with males having higher predicted FER total scores than females in Italy, Colombia, Germany, and Peru (Figure 2). The model revealed a significant interaction between education and country. While several countries showed a clear positive association between education and FER performance (e.g., Peru, Colombia), others exhibited no effect (e.g., Germany, Mexico).

#### 3.2.2 Presymptomatic pathogenic variant carriers

In presymptomatic carriers, the likelihood ratio test statistic between the model with country as random effect and the model comprising solely age, sex, and education was also significant [X^2^(1) = 35.95, *p* < 0.001], indicating superior prediction of scores when incorporating country in the model. The VPC indicated that 18.0% of the variance in FER performance can be attributed to differences among countries after controlling for age, sex, and education (Figure 1). The Hedges’ *g* ranged from *g* = 0.01 (Sweden vs. Belgium) to *g* = 1.17 (Netherlands vs. Germany). Variance across countries for each emotion (mean and 95% CI) is illustrated in Figure S2. The emotion with the largest country effect after controlling for age, sex, education, and pathogenic variant was surprise, VPC = 21.20%, followed by disgust, VPC = 15.18%, fear, VPC = 9.63%, anger, VPC = 8.20%, sadness, VPC = 2.67%, and happiness, VPC = 0.00%. When including language as a random effect in the model instead of country, we observe a lower VPC of 17.6%.

**FIGURE 3:**
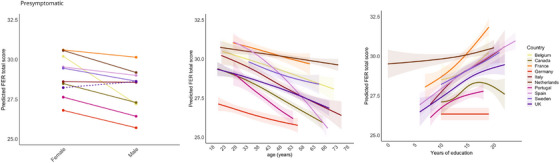
Effects of sex, age, and education per country in presymptomatic pathogenic variant carriers. Note: Dotted lines indicate a higher predicted FER total score in males compared to females. The effects of age and education on the predicted FER total score are visualized with 95% confidence intervals. FER, facial emotion recognition.

Age had a negative effect on the FER total score [β = −0.06, *p* < 0.01]. We also found a positive effect of education [β = 0.17, *p* < 0.01] and an effect of sex [β = −0.73, *p* < 0.001], with women having higher scores than men (Figure 3). No significant effects of pathogenic variant and language were found. Country interacted significantly with age and education on FER performance. For age, all countries show a negative predicted effect on FER performance, but some countries (e.g., Portugal, Spain) have stronger negative effects than others (e.g., Netherlands, United Kingdom) (Figure 3). For education, several countries showed a strong positive effect on FER performance (e.g., France, Spain), while other countries showed a less strong or even non‐significant effect (e.g., Germany, Portugal).

#### 3.2.3 Individuals with bvFTD

In individuals with bvFTD, the likelihood ratio test was significant [X^2^(1) = 5.40, *p* = 0.02], indicating superior prediction of scores when incorporating the random country effect into the model. The VPC indicated that 9.9% of the variance in FER performance can be attributed to differences among countries after controlling for age, sex, and education (Figure 1). Hedges’ *g* ranged from *g* = 0.01 (United Kingdom vs. Germany) to *g* = 1.28 (Colombia vs. Spain). Variances across countries for each emotion (mean and 95% CI) are illustrated in Figure S3. The emotion with the largest country effect after controlling for age, sex, education, and pathogenic variant was disgust, VPC = 13.31%, followed by sadness, VPC = 8.70%, surprise, VPC = 6.19%, anger, VPC = 4.74%, fear, VPC = 3.68%, and happiness, VPC = 0.00%. When including language as a random effect in the model instead of country, we observe a lower VPC of 2.9%.

**FIGURE 4:**
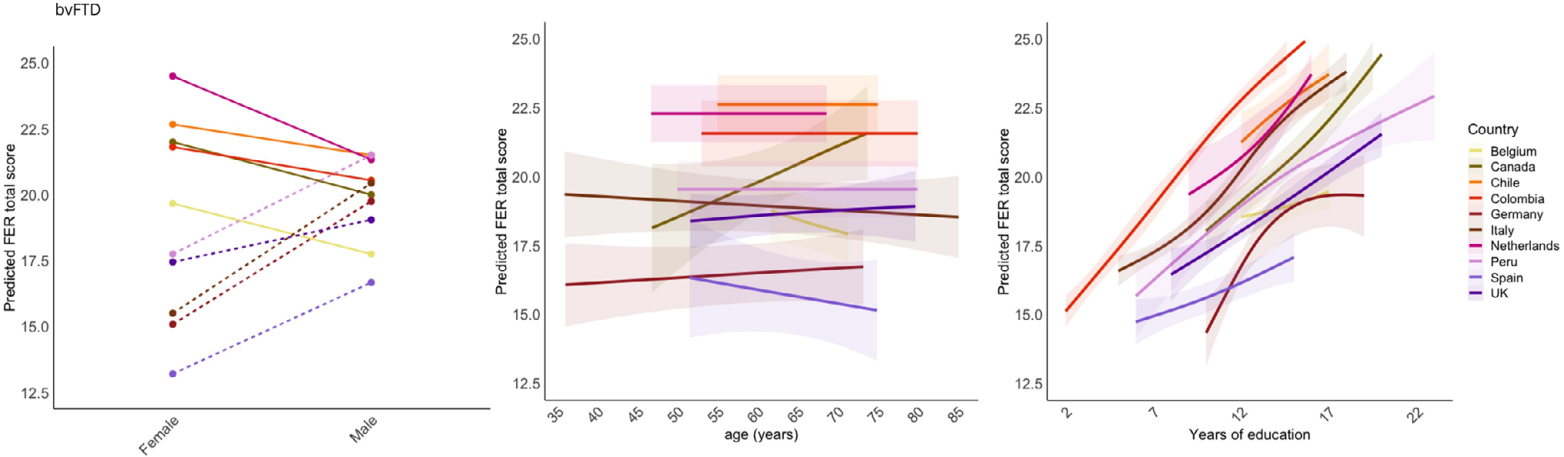
Effects of sex, age, and education per country in symptomatic pathogenic variant carriers. *Note*: Dotted lines indicate a higher predicted FER total score in males compared to females. The effects of age and education on the predicted FER total score are visualized with 95% confidence intervals. FER, facial emotion recognition.

Independent of country, we found a positive effect of education [β = 0.63, *p* <#x000A0;0.001]. No significant effects were found for age, sex, and language (Figure 4). However, country did interact with sex on FER performance, where males from Peru, Spain, Italy, Germany, and the United Kingdom had a higher predicted FER total score compared to females. All countries showed a positive predictive effect of education on FER performance. There was a significant interaction effect between country and education on FER performance. Some countries (e.g., Colombia, Canada) showed stronger positive effects than others (e.g., Spain, Belgium) (Figure 4).

### 3.3 Neuroanatomical correlates

**FIGURE 5:**
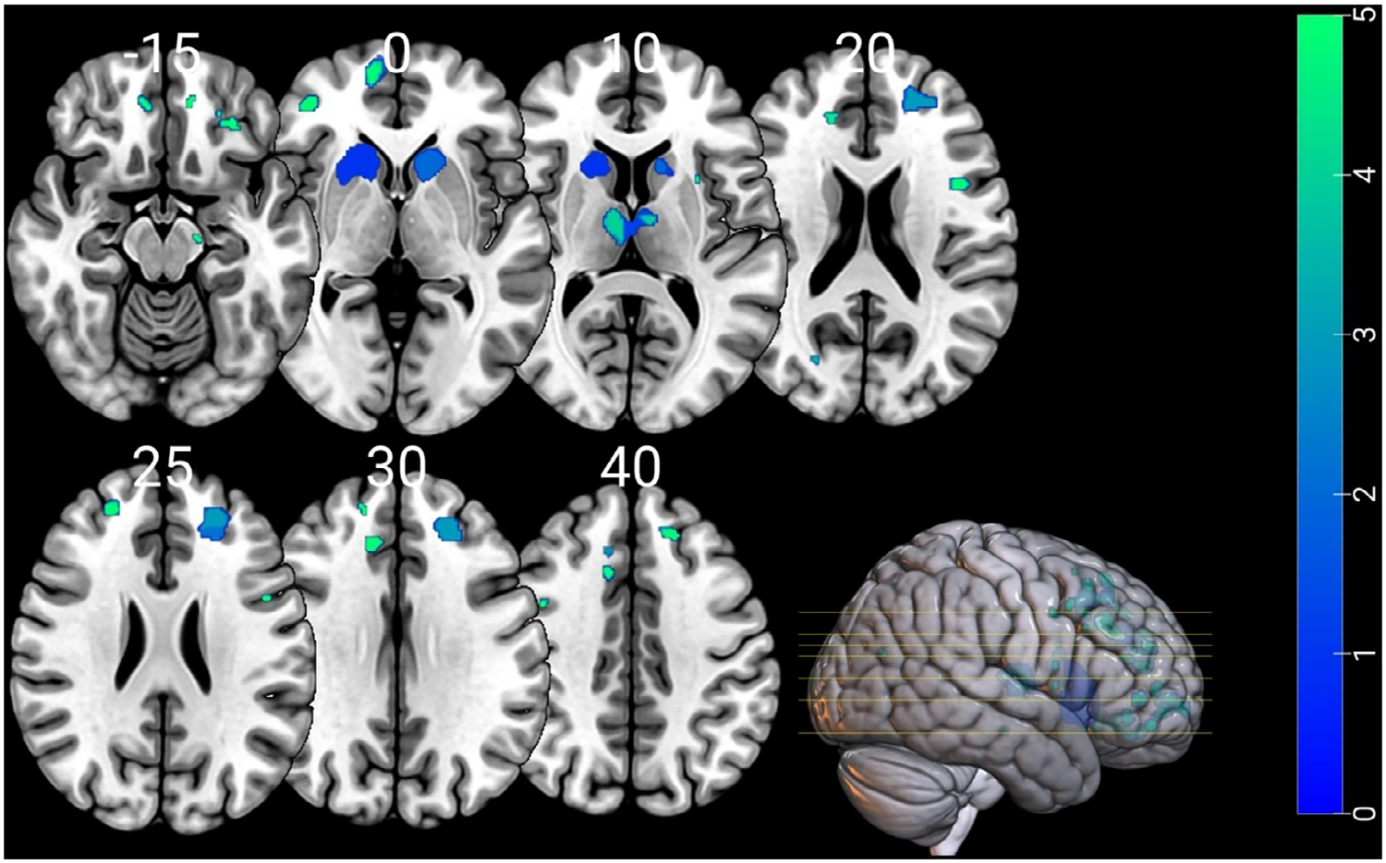
Neuroanatomical correlates of performance on the FER total score in individuals with bvFTD. Results are corrected for family‐wise error (*p* < 0.05). bvFTD, behavioral variant of frontotemporal dementia; FER, facial emotion recognition.

The VBM analyses showed no significant (FWE‐corrected) association between GM volume and FER performance in presymptomatic pathogenic variant carriers and controls. In individuals with bvFTD, the VBM analysis showed a significant association between FER performance and bilateral frontal areas (i.e., ventromedial prefrontal cortex, orbitofrontal cortex) and regions in the basal ganglia (i.e., caudate, putamen). A lower FER performance was associated with lower GM volume of the abovementioned regions (Figure 5). The interaction effect between country and FER on GM volume was not significant, indicating that no differences in brain signatures of FER were found across the different countries.

## 4 DISCUSSION

This study examined the influence of country on FER performance across FTD disease stages in 16 different countries. Results suggest that country is not only associated with FER performance but also moderates the effects of age, education, and sex. The association of GM volume and FER remained consistent across countries. These results shed light on why early deficits in social cognition have not been consistently observed in presymptomatic carriers of FTD pathogenic variants in global study cohorts. Furthermore, they emphasize the importance of re‐evaluating traditional neuropsychological methods and their applicability across cultural contexts in clinical settings and multicenter trials.

In all clinical groups, country was associated with FER performance. The proportion of variance explained by country on FER was 10% in individuals with bvFTD and almost 20% in controls and presymptomatic pathogenic variant carriers. A lower percentage of variance in individuals with bvFTD may reflect the degenerative nature of FTD, which could overshadow any moderating effects of country. Although there is less variance in bvFTD, it remains substantially higher than what has been observed across countries in other cognitive domains, such as memory^32^ and spatial navigation (both VPCs are 1.7%).^33^ This finding suggests that cultural influences shape social cognition from the preclinical stage through the progression of the disease. Factors such as cultural norms and societal attitudes likely contribute to this effect.^17^, ^20^, ^24^, ^34^, ^35^ This highlights that country has a significant impact even in patient populations, complicating cross‐country analysis of social cognition.

In our study, the observed variations between countries cannot be attributed to differences in language or translation, which is in line with the study of Quesque et al.^24^ Cross‐country analyses that controlled for the language of administration revealed no significant impact on FER performance. Furthermore, including language as a random effect in the model resulted in a reduction in variance. This outcome was expected, given that data from multiple countries sharing the same language were aggregated. These findings suggest that the observed differences in FER performance across countries are likely due to other contextual or cultural factors, rather than linguistic influences.

The percentage of variance found in our control group was consistent with the variance found in the study of Quesque et al.^24^ However, we observed different effect sizes of the overlapping countries. For example, Quesque et al.^24^ found a significant difference in effect size between the United Kingdom and Spain, whereas in our study, this difference is reversed, with Spain outperforming the United Kingdom. In addition, in the current study, the variations in effect sizes between countries were not uniform across the clinical groups. For example, while Spanish controls demonstrated relatively strong performance on FER, presymptomatic variant carriers from Spain performed worse compared to those in other countries. This implies that country is not the only influencing factor; other moderating variables, such as sample size, demographic characteristics (e.g., education level, age, sex, but also socioeconomic status), and recruitment strategies, also influence FER performance. In the study of Quesque et al.,^24^ healthy participants were older with fewer years of education. Our control group is comparable with the presymptomatic and symptomatic carrier group as, they are first‐degree pathogenic‐negative family members (i.e., GENFI). Nonetheless, these findings imply that country effects on FER are complex and may interact with other factors,^24^ which raises concerns about the replicability of our findings.

We found a significant effect of education on FER performance in all clinical groups: Independent of the country of the participants, individuals with higher education tend to perform better on tests for FER.^36^, ^37^ However, this contrasts with Quesque et al.,^24^ finding no effect of education and suggesting that the effect of education on FER might vary across countries. Indeed, some studies regarding FER find effects of education, such as in France,^38^ but others do not find significant contributions of education on FER, such as in the Netherlands on the Facial Expressions of Emotion‐Stimuli and Tests (FEEST) (based on the Ekman 60 faces test).^39^ Similarly, our study found an interaction effect of country and education on predicted FER performance, with significant effects observed in some countries but minimal or no effects in others. Possible methodological factors, such as generally lower FER performance in certain countries and reduced variability in FER total scores and/or education level, may have contributed to this. However, this could also suggest that educational differences and schooling systems vary between cultures, which could explain differences in FER performance between countries that are geographically close to one another. This underscores that demographic effects on performance on the FER test are not consistent across countries, highlighting that norm scores established in one context cannot simply be applied to other countries.

There was no effect of age on FER performance in healthy controls and individuals with bvFTD, which is in line with other studies performed in controls^38^, ^39^ and individuals with bvFTD.^40^ In presymptomatic carriers, we found a significant effect of age, where older individuals perform worse on FER. A factor contributing to this effect could be that older individuals in the presymptomatic group are closer to disease onset and therefore have a lower FER performance.^5^ Furthermore, there was a significant interaction effect between country and age on FER performance. This suggests that age‐related changes in FER performance vary across populations.

Consistent with previous studies, we found significant sex differences in FER performance among healthy controls and presymptomatic carriers, with females performing better than males.^5^, ^24^, ^41^ Interestingly, in individuals with bvFTD, sex did not have a significant contribution to the model after correcting for the effect of country. This finding contradicts the study by de Boer et al.^42^ suggesting that females have a higher social cognition reserve. Moreover, we observe that in half of the countries, males perform higher on FER compared to females. Taken together, the variability in the relationship between country and demographic factors emphasizes the importance of cross‐cultural research in understanding how educational systems, socioeconomic factors, gender roles, and social norms and rules interact across cultures to shape social cognition.

Similar to previous studies,^24^, ^43^ variance in FER performance between countries was least prominent in what can be considered positive emotions (happy and surprise) and more prominent in what can be considered negative emotions (fear, anger, disgust, sadness). The recognition of negative emotions seems to be more culture‐dependent, which could be related to cultural rules.^44^, ^45^ In addition, most items of the FER test are negative, potentially causing higher variations in performance on these emotions compared to happy emotions.^24^ However, when comparing presymptomatic carriers to controls, we noted greater variance in the recognition of surprise, driven primarily by a lower performance in Germany and Spain. This discrepancy may arise from ambiguity between surprise and fear facial expressions in these countries.^24^ Moreover, according to Zhao et al.,^46^ there are neural mechanisms that underlie both fearful and surprised faces, including the parahippocampal gyrus and the amygdala in the limbic system. As reported by Bochetta et al.,^47^ the amygdala and hippocampus are significantly smaller in presymptomatic *C9orf72* and *MAPT* pathogenic variant carriers. The variance in surprise found in presymptomatic carriers may therefore be a potential marker for disease onset.

Regarding brain correlates in our study, VBM results revealed a significant association between FER performance and GM volume in individuals with bvFTD, specifically in bilateral frontal regions (ventromedial prefrontal cortex, orbitofrontal cortex) and subcortical structures (caudate and putamen). These findings align with the established role of these regions in social and emotional processing.^48^, ^49^ Interaction terms of country and FER performance were included in the analyses; however, these effects were not significant, suggesting the structural basis of FER deficits in bvFTD is consistent across countries. Therefore, the neuroanatomical underpinnings of FER deficits in bvFTD are robust and are not heavily influenced by cultural variations in emotion perception. This supports the notion that structural brain changes in these regions are key biomarkers for social cognition impairments in bvFTD, regardless of cultural context.

The major strength of this study is the large cohort of genetic FTD pathogenic variant carriers and individuals with bvFTD, paired with a well‐matched control group of pathogenic‐negative family members in the GENFI cohort. Nonetheless, in the ReDLat cohort, individuals with bvFTD did not undergo genetic testing, resulting in omission of potential genetic factors in these participants. In addition, despite the relatively large numbers for a rare condition, sample sizes per country were small and predominantly composed of participants from Western Europe and Latin America, limiting generalizability to other regions. Future studies should include more data from different countries, as well as measures of culture, as an individual's cultural background could not match their geographical location.

In conclusion, the findings of the current study emphasize the need to account for national and cultural differences in social cognition in FTD. We highlight the complex interplay of country on the general effects of age, sex, and education on FER, suggesting these factors cannot be assumed to be uniform across all countries. Currently, the use of the FER test in multicenter research requires careful consideration of cultural factors, and it remains unclear whether more culture‐sensitive assessments can detect early decline in FTD. Clinically, this emphasizes the importance of considering cultural factors when evaluating social cognition in individuals with FTD, as these factors may impact diagnosis, prognosis, and the tailoring of interventions for individuals from diverse cultural backgrounds.

## CONFLICT OF INTEREST STATEMENT

The authors declare no conflicts of interest. Author disclosures are available in the Supporting Information.

## CONSENT STATEMENT

All GENFI and ReDLat sites had local ethical approval for the study and all participants provided written informed consent.

## Supporting information



Supporting information

Supporting information

## Collaborators

**Table:**

Rhian Convery	Department of Neurodegenerative Disease, Dementia Research Centre, UCL Queen Square Institute of Neurology, London, UK
Martina Bocchetta	Department of Neurodegenerative Disease, Dementia Research Centre, UCL Queen Square Institute of Neurology, London, UK
David Cash	Department of Neurodegenerative Disease, Dementia Research Centre, UCL Queen Square Institute of Neurology, London, UK
Sophie Goldsmith	Department of Neurodegenerative Disease, Dementia Research Centre, UCL Queen Square Institute of Neurology, London, UK
Kiran Samra	Department of Neurodegenerative Disease, Dementia Research Centre, UCL Queen Square Institute of Neurology, London, UK
David L. Thomas	Neuroimaging Analysis Centre, Department of Brain Repair and Rehabilitation, UCL Institute of Neurology, Queen Square, London, UK
Thomas Cope	Cambridge University Hospitals NHS Trust, Cambridge UK
Maura Malpetti	Department of Clinical Neurosciences, University of Cambridge, Cambridge, UK
Antonella Alberici	Centre for Neurodegenerative Disorders, Department of Clinical and Experimental Sciences, University of Brescia, Brescia, Italy
Enrico Premi	Stroke Unit, ASST Brescia Hospital, Brescia, Italy
Roberto Gasparotti	Neuroradiology Unit, University of Brescia, Brescia, Italy
Emanuele Buratti	ICGEB Trieste, Italy
Valentina Cantoni	Centre for Neurodegenerative Disorders, Department of Clinical and Experimental Sciences, University of Brescia, Brescia, Italy
Andrea Arighi	Fondazione IRCCS Ca’ Granda Ospedale Maggiore Policlinico, Neurodegenerative Diseases Unit, Milan, Italy
Chiara Fenoglio	University of Milan, Centro Dino Ferrari, Milan, Italy
Vittoria Borracci	Fondazione IRCCS Ca’ Granda Ospedale Maggiore Policlinico, Neurodegenerative Diseases Unit, Milan, Italy
Maria Serpente	Fondazione IRCCS Ca’ Granda Ospedale Maggiore Policlinico, Neurodegenerative Diseases Unit, Milan, Italy
Tiziana Carandini	Fondazione IRCCS Ca’ Granda Ospedale Maggiore Policlinico, Neurodegenerative Diseases Unit, Milan, Italy
Emanuela Rotondo	Fondazione IRCCS Ca’ Granda Ospedale Maggiore Policlinico, Neurodegenerative Diseases Unit, Milan, Italy
Giacomina Rossi	Fondazione IRCCS Istituto Neurologico Carlo Besta, Milano, Italy
Giorgio Giaccone	Fondazione IRCCS Istituto Neurologico Carlo Besta, Milano, Italy
Giuseppe Di Fede	Fondazione IRCCS Istituto Neurologico Carlo Besta, Milano, Italy
Paola Caroppo	Fondazione IRCCS Istituto Neurologico Carlo Besta, Milano, Italy
Sara Prioni	Fondazione IRCCS Istituto Neurologico Carlo Besta, Milano, Italy
Veronica Redaelli	Fondazione IRCCS Istituto Neurologico Carlo Besta, Milano, Italy
David Tang‐Wai	The University Health Network, Krembil Research Institute, Toronto, Canada
Ekaterina Rogaeva	Tanz Centre for Research in Neurodegenerative Diseases, University of Toronto, Toronto, Canada
Johanna Krüger	Research Unit of Clinical Medicine, Neurology, University of Oulu, Oulu, Finland
Miguel Castelo‐Branco	Faculty of Medicine, ICNAS, CIBIT, University of Coimbra, Coimbra, Portugal.
Morris Freedman	Baycrest Health Sciences, Rotman Research Institute, University of Toronto, Toronto, Canada
Ron Keren	The University Health Network, Toronto Rehabilitation Institute, Toronto, Canada
Sandra Black	Sunnybrook Health Sciences Centre, Sunnybrook Research Institute, University of Toronto, Toronto, Canada
Sara Mitchell	Sunnybrook Health Sciences Centre, Sunnybrook Research Institute, University of Toronto, Toronto, Canada
Christen Shoesmith	Department of Clinical Neurological Sciences, University of Western Ontario, London, Ontario, Canada
Robart Bartha	Department of Medical Biophysics, The University of Western Ontario, London, Ontario, Canada
Rosa Rademakers	Center for Molecular Neurology, University of Antwerp
Jackie Poos	Department of Neurology, Erasmus Medical Center, Rotterdam, Netherlands
Janne M. Papma	Department of Neurology, Erasmus Medical Center, Rotterdam, Netherlands
Lucia Giannini	Department of Neurology, Erasmus Medical Center, Rotterdam, Netherlands
Liset de Boer	Department of Neurology, Erasmus Medical Center, Rotterdam, Netherlands
Julie de Houwer	Department of Neurology, Erasmus Medical Center, Rotterdam, Netherlands
Rick van Minkelen	Department of Clinical Genetics, Erasmus Medical Center, Rotterdam, Netherlands
Yolande Pijnenburg	Amsterdam University Medical Centre, Amsterdam VUMC, Amsterdam, Netherlands
Benedetta Nacmias	Department of Neuroscience, Psychology, Drug Research and Child Health, University of Florence, Florence, Italy
Camilla Ferrari	Department of Neuroscience, Psychology, Drug Research and Child Health, University of Florence, Florence, Italy
Cristina Polito	Department of Biomedical, Experimental and Clinical Sciences “Mario Serio”, Nuclear Medicine Unit, University of Florence, Florence, Italy
Gemma Lombardi	Department of Neuroscience, Psychology, Drug Research and Child Health, University of Florence, Florence, Italy
Valentina Bessi	Department of Neuroscience, Psychology, Drug Research and Child Health, University of Florence, Florence, Italy
Enrico Fainardi	Neuroradiology Unit, Department of Experimental and Clinical Biomedical Sciences, University of Florence, Florence, Italy
Stefano Chiti	Neuroradiology Unit, Department of Experimental and Clinical Biomedical Sciences, University of Florence, Florence, Italy
Mattias Nilsson	Department of Clinical Neuroscience, Karolinska Institutet, Stockholm, Sweden
Henrik Viklund	Karolinska University Hospital Huddinge
Melissa Taheri Rydell	Department of Neurobiology, Care Sciences and Society; Center for Alzheimer Research, Division of Neurogeriatrics, Bioclinicum, Karolinska Institutet, Solna, Sweden
Vesna Jelic	Department of Neurobiology, Care Sciences and Society; Division of Clinical Geriatrics, Karolinska Institutet, Stockholm, Sweden
Abbe Ullgren	Department of Neurobiology, Care Sciences and Society, Center for Alzheimer Research, Division of Neurogeriatrics, Bioclinicum, Karolinska Institutet, Solna, Sweden
Elena Rodriguez‐Vieitez	Department of Neurobiology, Care Sciences and Society, Center for Alzheimer Research, Division of Neurogeriatrics, Bioclinicum, Karolinska Institutet, Solna, Sweden
Tobias Langheinrich	Division of Neuroscience and Experimental Psychology, Wolfson Molecular Imaging Centre, University of Manchester, Manchester, UK
Albert Lladó	Alzheimer's disease and Other Cognitive Disorders Unit, Neurology Service, Hospital Clínic, Barcelona, Spain
Anna Antonell	Alzheimer's disease and Other Cognitive Disorders Unit, Neurology Service, Hospital Clínic, Barcelona, Spain
Jaume Olives	Alzheimer's disease and Other Cognitive Disorders Unit, Neurology Service, Hospital Clínic, Barcelona, Spain
Mircea Balasa	Alzheimer's disease and Other Cognitive Disorders Unit, Neurology Service, Hospital Clínic, Barcelona, Spain
Nuria Bargalló	Imaging Diagnostic Center, Hospital Clínic, Barcelona, Spain
Sergi Borrego‐Ecija	Alzheimer's disease and Other Cognitive Disorders Unit, Neurology Service, Hospital Clínic, Barcelona, Spain
Ana Verdelho	Department of Neurosciences and Mental Health, Centro Hospitalar Lisboa Norte—Hospital de Santa Maria & Faculty of Medicine, University of Lisbon, Lisbon, Portugal
Carolina Maruta	Laboratory of Language Research, Centro de Estudos Egas Moniz, Faculty of Medicine, University of Lisbon, Lisbon, Portugal
Tiago Costa‐Coelho	Faculty of Medicine, University of Lisbon, Lisbon, Portugal
Gabriel Miltenberger	Faculty of Medicine, University of Lisbon, Lisbon, Portugal
Frederico Simões do Couto	Faculdade de Medicina, Universidade Católica Portuguesa
Alazne Gabilondo	Cognitive Disorders Unit, Department of Neurology, Donostia University Hospital, San Sebastian, Gipuzkoa, Spain
Ioana Croitoru	Instituto de Investigación Sanitaria Biogipuzkoa, Neurosciences Area, Group of Neurodegenerative Diseases, San Sebastian, Spain.
Mikel Tainta	Instituto de Investigación Sanitaria Biogipuzkoa, Neurosciences Area, Group of Neurodegenerative Diseases, San Sebastian, Spain.
Myriam Barandiaran	Cognitive Disorders Unit, Department of Neurology, Donostia University Hospital, San Sebastian, Gipuzkoa, Spain
Patricia Alves	Instituto de Investigación Sanitaria Biogipuzkoa, Neurosciences Area, Group of Neurodegenerative Diseases, San Sebastian, Spain.
Benjamin Bender	Department of Diagnostic and Interventional Neuroradiology, University of Tübingen, Tübingen, Germany
David Mengel	Department of Neurodegenerative Diseases, Hertie‐Institute for Clinical Brain Research and Center of Neurology, University of Tübingen, Tübingen, Germany
Lisa Graf	Department of Neurodegenerative Diseases, Hertie‐Institute for Clinical Brain Research and Center of Neurology, University of Tübingen, Tübingen, Germany
Annick Vogels	Department of Human Genetics, KU Leuven, Leuven, Belgium
Mathieu Vandenbulcke	Geriatric Psychiatry Service, University Hospitals Leuven, Belgium; Neuropsychiatry, Department of Neurosciences, KU Leuven, Leuven, Belgium
Philip Van Damme	Neurology Service, University Hospitals Leuven, Belgium; Laboratory for Neurobiology, VIB‐KU Leuven Centre for Brain Research, Leuven, Belgium
Rose Bruffaerts	Department of Biomedical Sciences, University of Antwerp, Antwerp, Belgium; Biomedical Research Institute, Hasselt University, 3500 Hasselt, Belgium
Koen Poesen	Laboratory for Molecular Neurobiomarker Research, KU Leuven, Leuven, Belgium
Pedro Rosa‐Neto	Translational Neuroimaging Laboratory, McGill Centre for Studies in Aging, McGill University, Montreal, Québec, Canada
Maxime Montembault	Douglas Research Centre, Department of Psychiatry, McGill University, Montreal, Québec, Canada
Raffaella Lara Migliaccio	Sorbonne Université, Paris Brain Institute – Institut du Cerveau – ICM, Inserm U1127, CNRS UMR 7225, AP‐HP—Hôpital Pitié‐Salpêtrière, Paris, France
Ninon Burgos	Sorbonne Université, Paris Brain Institute – Institut du Cerveau – ICM, Inserm U1127, CNRS UMR 7225, AP‐HP—Hôpital Pitié‐Salpêtrière, Paris, France
Daisy Rinaldi	Sorbonne Université, Paris Brain Institute – Institut du Cerveau – ICM, Inserm U1127, CNRS UMR 7225, AP‐HP—Hôpital Pitié‐Salpêtrière, Paris, France
Catharina Prix	Neurologische Klinik, Ludwig‐Maximilians‐Universität München, Munich, Germany
Elisabeth Wlasich	Neurologische Klinik, Ludwig‐Maximilians‐Universität München, Munich, Germany
Olivia Wagemann	Neurologische Klinik, Ludwig‐Maximilians‐Universität München, Munich, Germany
Sonja Schönecker	Neurologische Klinik, Ludwig‐Maximilians‐Universität München, Munich, Germany
Alexander Maximilian Bernhardt	Neurologische Klinik, Ludwig‐Maximilians‐Universität München, Munich, Germany
Anna Stockbauer	Neurologische Klinik, Ludwig‐Maximilians‐Universität München, Munich, Germany
Jolina Lombardi	Department of Neurology, University of Ulm, Ulm
Sarah Anderl‐Straub	Department of Neurology, University of Ulm, Ulm, Germany
Adeline Rollin	CHU, CNR‐MAJ, Labex Distalz, LiCEND Lille, France
Gregory Kuchcinski	Univ Lille, France; Inserm 1172, Lille, France; CHU, CNR‐MAJ, Labex Distalz, LiCEND Lille, France
Vincent Deramecourt	Univ Lille, France; Inserm 1172, Lille, France; CHU, CNR‐MAJ, Labex Distalz, LiCEND Lille, France
João Durães	Neurology Department, Centro Hospitalar e Universitario de Coimbra, Coimbra, Portugal
Marisa Lima	Neurology Department, Centro Hospitalar e Universitario de Coimbra, Coimbra, Portugal
Maria João Leitão	Centre of Neurosciences and Cell Biology, Universidade de Coimbra, Coimbra, Portugal
Maria Rosario Almeida	Faculty of Medicine, University of Coimbra, Coimbra, Portugal
Miguel Tábuas‐Pereira	Neurology Department, Centro Hospitalar e Universitario de Coimbra, Coimbra, Portugal; Faculty of Medicine, University of Coimbra, Coimbra, Portugal.
Sónia Afonso	Instituto Ciencias Nucleares Aplicadas a Saude, Universidade de Coimbra, Coimbra, Portugal
João Lemos	Faculty of Medicine, University of Coimbra, Coimbra, Portugal
