# Supplementary material for: Cross‐country variance in facial emotion recognition in presymptomatic and symptomatic behavioral variant frontotemporal dementia: Insights from the GENFI and ReDLat consortia
Source: Alzheimers Dement. 2025 Oct 14;21(10):e70741. doi: 10.1002/alz.70741 (PMC12519523; doi:10.1002/alz.70741)

**Supplementary Table 1.** Demographic data and mean FER performance per clinical group in the GENFI and ReDLat cohort.

|  | **BvFTD**  **(n=159)** | **Presymptomatic (n=421)** | **Controls**  **(n=583)** | **Statistical differences** |
| --- | --- | --- | --- | --- |
| **GENFI COHORT (n=884)** | | | | |
| N | 107 | 421 | 356 |  |
| Pathogenic variant | 62 C9orf72  20 GRN  24 MAPT  1 TARDBP | 182 C9orf72  166 GRN  66 MAPT  5 TBK  1 TARDBP  1 VCP | N/A | N/A |
| Sex ratio (female) | 36.45% | 58.91% | 57.58% | significant |
| Age | 62.27 (8.81) | 44.30 (11.66) | 45.37 (12.72) | bvFTD > pre, con |
| Education (years) | 12.63 (3.66) | 14.73 (3.37) | 14.77 (3.28) | bvFTD < pre, con |
| FER total score | 18.91 (6.27) | 28.75 (3.21) | 28.79 (3.18) | bvFTD < pre, con |
| **REDLAT COHORT (n=279)** | | | | |
| N | 52 | N/A | 277 |  |
| Sex ratio (female) | 50.00% | N/A | 71.81% | significant |
| Age | 66.92 (8.04) | N/A | 60.89 (12.25) | bvFTD < con |
| Education | 13.06 (4.73) | N/A | 13.70 (5.38) | Ns. |
| FER total score | 21.67 (6.42) | N/A | 26.17 (4.65) | bvFTD < con |

*Note*. Data are presented as mean (standard deviation). Abbreviations: *C9orf72,* chromosome 9 open reading frame 72; *GRN*, progranulin; *MAPT*, microtubule-associated protein tau; *TARDP, TAR-DNA-binding protein*; *TBK*, TANK-binding kinase; VCP, valosin-containing protein FER, Facial Emotion Recognition.

**Supplementary figure 1.** Mean and 95% CI of FER subscores per country in healthy controls.


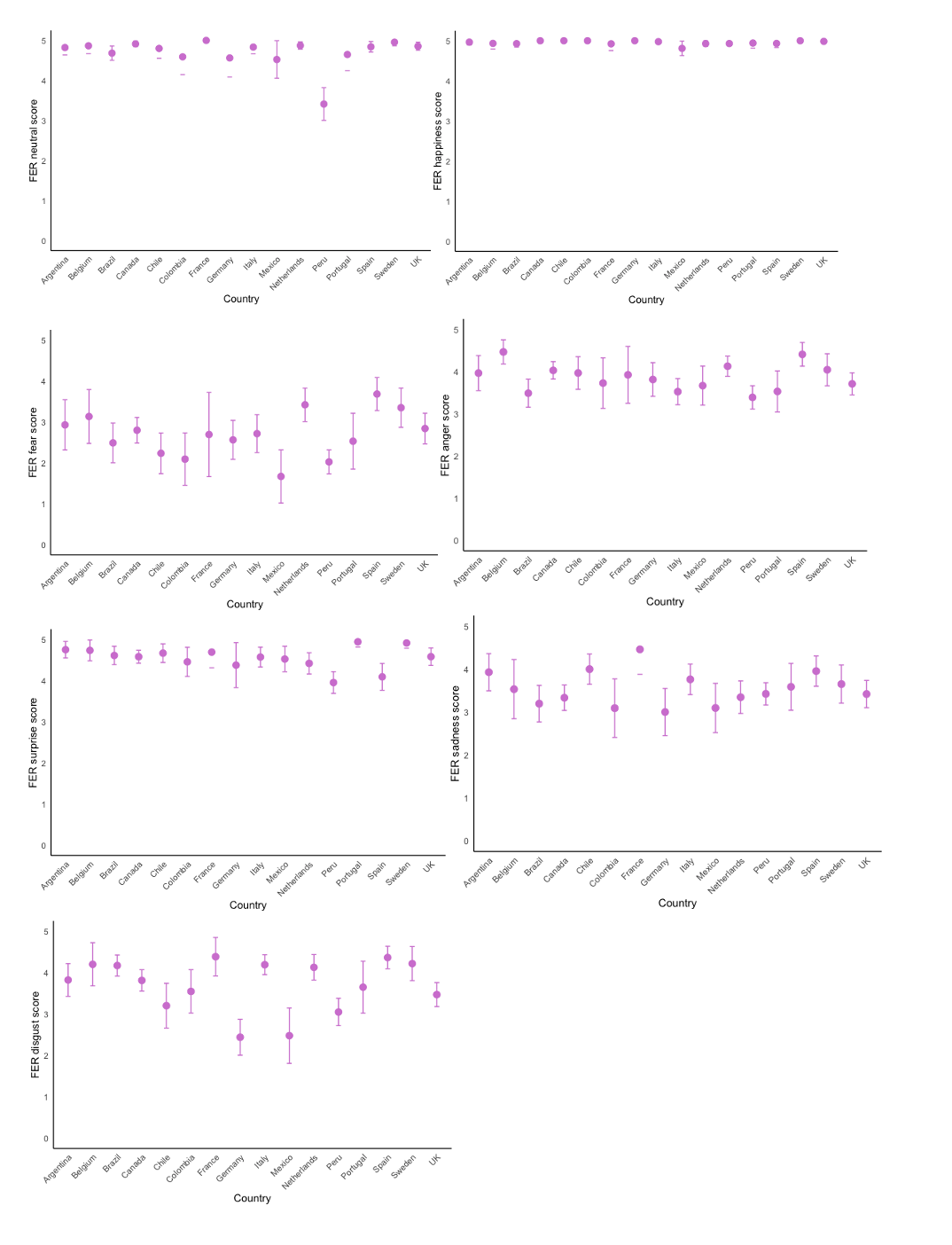


**Supplementary figure 2.** Mean and 95% CI of FER subscores per country in presymptomatic carriers.


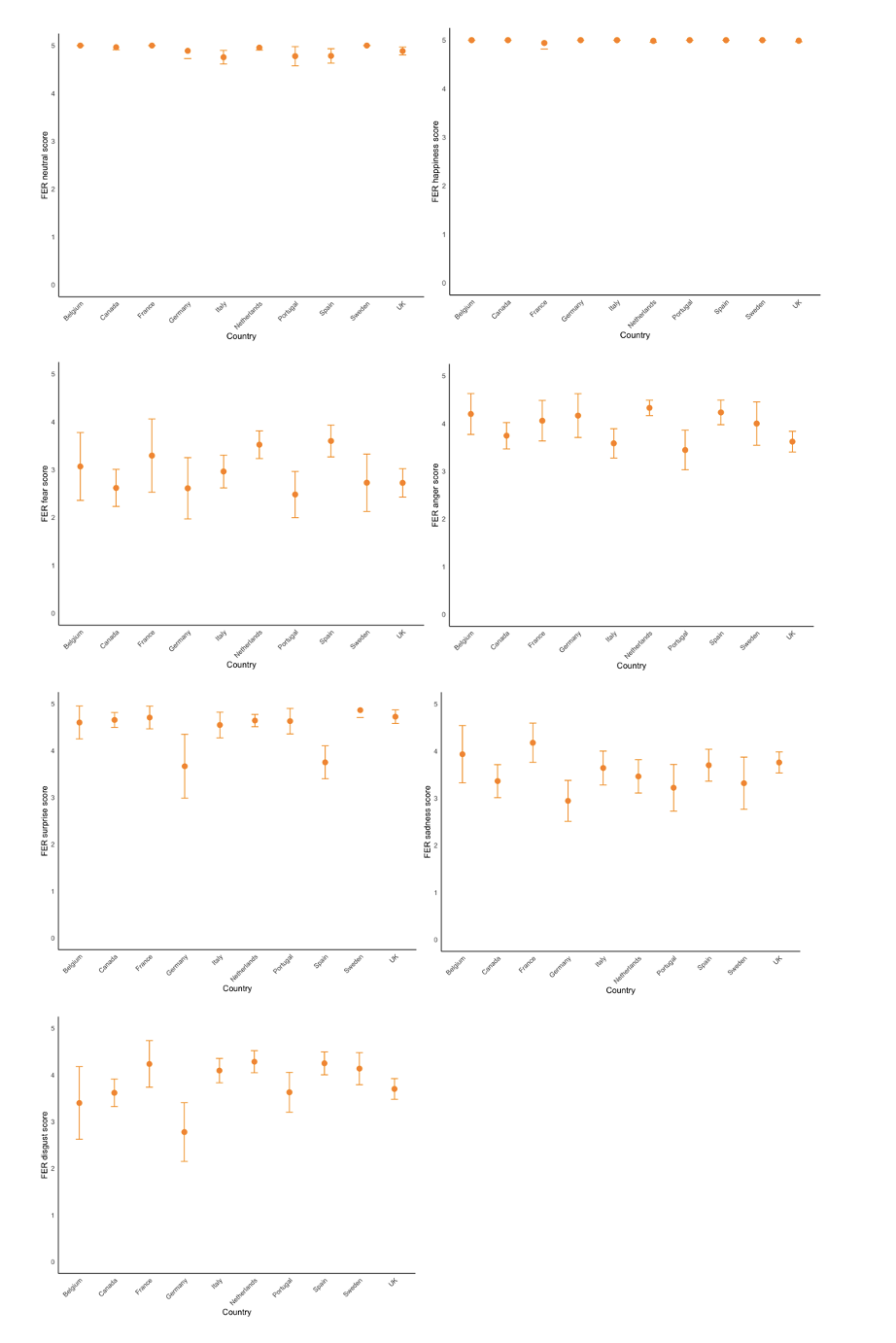


**Supplementary figure 3.** Mean and 95% CI of FER subscores per country in individuals with bvFTD.


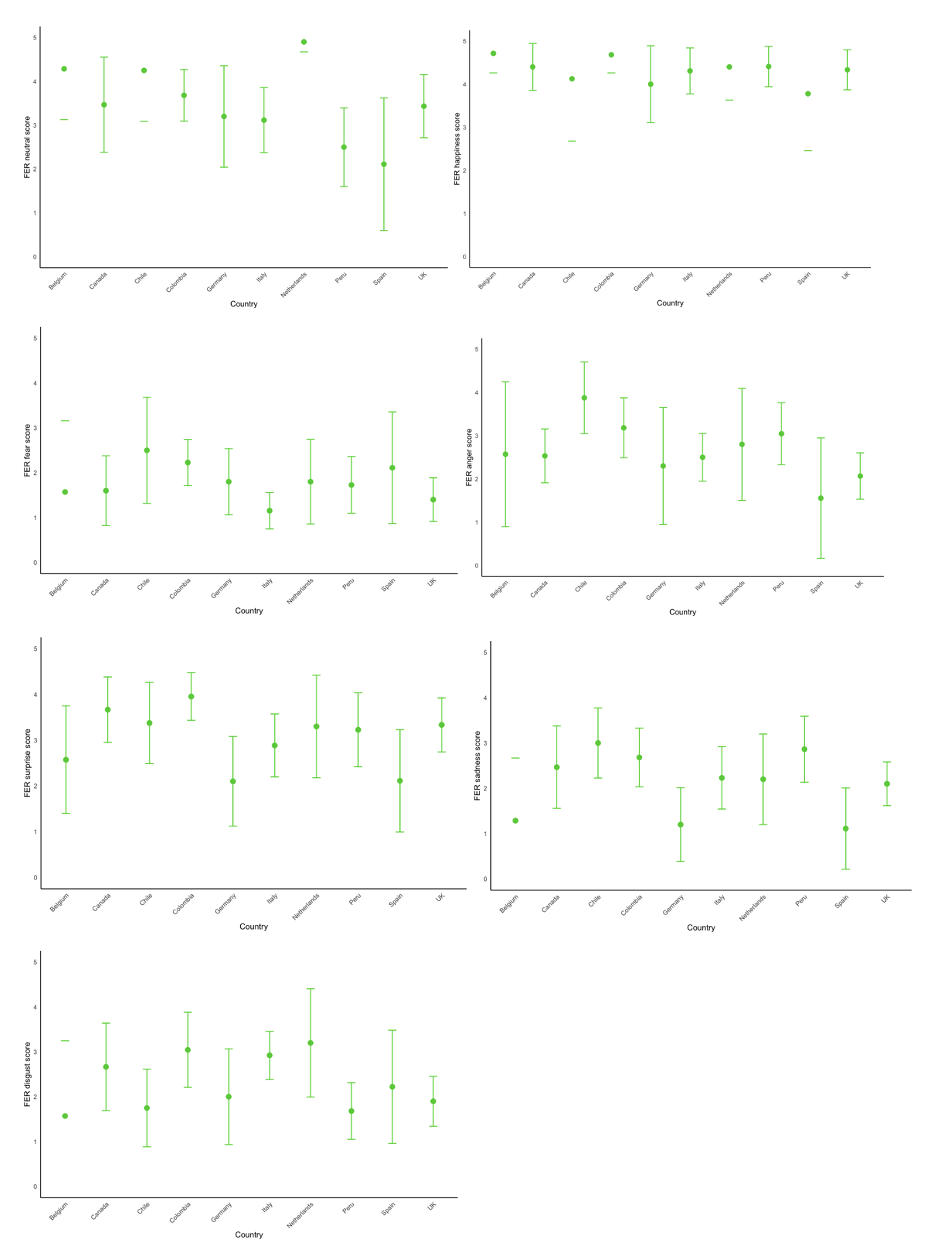

Supplement: Supplementary file 1 — Supporting information [file ALZ-21-e70741-s001.docx]
